# What are the psychological correlates of perceived adverse food reactions?

**DOI:** 10.1186/2045-7022-1-S1-P116

**Published:** 2011-08-12

**Authors:** Saliha Mahmood

**Affiliations:** 1 Kings College London, Ickehnam, UK

## Background

Population studies suggest that around 20.4% of the UK population consider themselves affected by perceived adverse food reactions. However, formal testing reveals that only 1.4% are truly affected by either food allergy and/or food intolerance. The reason for this large discrepancy has been the subject of very few studies. This study aims to identify the prevalence of perceived adverse food reactions (PAFR) in a healthy population, and to evaluate any relationship between PAFR and psychological factors such as disordered eating, subjective stress and modern health worries.

## Methods

A self report questionnaire was completed by 194 medical or physiotherapy students. Overall response rate was 80.8%. The questionnaire identified individuals with a PAFR and used validated measures of bulimic symptomatology (Bulimic Inventory Test Edinburgh), subjective stress (Perceived Stress Scale) and modern health worries (Modern Health Worries Scale).

## Results

The prevalence of PAFR was found to be 28.4%. Female and physiotherapy students were found to be more likely to report food reactions than male or medical students. Those participants who had a PAFR demonstrated higher levels of bulimic symptomatology (p< 0.0001) and higher levels of subjective stress than controls (p<0.05). Levels of modern health worries did not differ significantly in participants with and without a PAFR.

## Conclusion

This study has made progress in validating a questionnaire to measure adverse food reactions. This instrument may be of benefit to future researchers and clinicians alike. This study suggests that PAFR’s are related to bulimic patterns of disordered eating and perceived stress levels. In light of this finding, healthcare professionals such as dieticians may find benefit in screening for bulimia nervosa in individuals presenting with a PAFR: particularly young, stressed females who may use adverse food reactions to justify maladaptive eating.

